# Haze Attitudes and the Willingness to Pay for Haze Improvement: Evidence from Four Cities in Shandong Province, China

**DOI:** 10.3390/ijerph15102297

**Published:** 2018-10-19

**Authors:** Fan Yang, Ling Ding, Cai Liu, Lizheng Xu, Stephen Nicholas, Jian Wang

**Affiliations:** 1 Center for Health Economics Experiment and Public Policy, School of Public Health, Shandong University, No. 44 Wenhuaxi Road, Lixia, District, Jinan 250012, China; 15153132375@163.com (F.Y.); sddxxlz@163.com (L.X.); 2 Tianjin Hongqiao District Health and Family Planning Commission, 202 Qinjian Road, Hongqiao 300131, China; wydl1222@163.com; 3 School of Management, Tianjin University of Traditional Chinese Medicine, Tianjin 300193, China; keidy0707@163.com; 4 School of Economics and School of Management, Tianjin Normal University, West Bin Shui Avenue, Tianjin 300074, China; 5 TOP Education Institute, 1 Central Avenue Australian Technology Park, Eveleigh Sydney, NSW 2015, Australia; 6 Newcastle Business School, University of Newcastle, University Drive, Newcastle, NSW 2308, Australia

**Keywords:** contingent valuation method, willingness to pay, haze governance, Shandong province

## Abstract

Background: Given the health and welfare impacts of haze, haze reduction governance challenges Chinese policy-makers. Surprisingly, there have been no studies of the differences in the public’s willingness to pay (WTP) for haze governance within a province. Yet haze reduction policies are implemented at the provincial level. Based on the contingent valuation method, data on WTP for haze governance across four industrial cities in Shandong province were collected using a questionnaire survey. Method: A combination of stratified sampling and non-probability sampling methods were used, yielding a valid sample of 1006 respondents. The Heckman sample selection model was used to analyze factors determining WTP and WTP amount. Results: 53% of respondents were unwilling to pay for haze reduction, while less than 1% of these respondents were satisfied with Shandong’s air quality. About half (47%) of the respondents were willing to pay, on average, US$14.14 per household per year for haze governance. We found that there were significant inter-city differences in the WTP and WTP amounts: those with a higher income, education, haze knowledge, and haze concern were WTP; age, marital status, and subjective indicators displayed a negative relationship with WTP amount. About two thirds of the non-payers, and those with poor environmental knowledge, argued that air quality improvement was mainly the responsibility of governments (39.3%) and polluters (25.6%), instead of ordinary citizens. Further, 27% of non-payers said that their income was too low to contribute to a pollution tax and 6.3% claimed that they did not believe the funds would be used effectively for environmental conservation. Conclusions: City-specific differences in WTP may caution against “one size fits all” policies. The study indicates that the government may need to target policies to specific cities and the characteristics of residents in those cities by age, education, and income groups and residents’ subjective evaluation of the government and the haze problem and those responsible for pollution.

## 1. Introduction

China’s rapid industrialization and urbanization have given rise to unprecedented environmental challenges, including haze and water pollution, soil erosion, sand storms, biodiversity loss, solid waste management problems, and acid rain [1,2,3,4]. Urban haze, driven mainly by fossil fuel use, especially coal and hydrocarbons [5,6], imposes significant costs on individuals and China’s health system. For example, in the Pearl River Delta Region that accounts for over 10% of China’s national gross domestic product (GDP), the total economic loss due to the health effects of PM_10_ (particulate matter whose diameter is below 10 μm) was estimated to be US$45 billion, equivalent to 1.35% of the regional economy’s GDP [7]. In haze-polluted environments, the World Health Organization estimated that respiratory disease caused the loss of 2.25 disability-adjusted life years per thousand people.

While pollution governance, in particular haze reduction, is the single most important focus of Chinese government environmental policy [8], haze reduction targets are frequently unmet, with 338 of China’s largest cities experiencing, on average, deteriorating air quality between 2016 and 2017 [9]. Improved quality of life and health outcomes from haze reduction are tied to the interplay between the efficacy of public environmental policies and the public’s willingness-to-pay (WTP) for haze governance. Considering the social and health harm of haze and the serious haze reduction burden, this paper measures the public’s attitudes towards improving haze weather and what factors matter for their WTP for haze reduction. Selecting four cities in one of China’s most haze prone provinces, the industrial province of Shandong, we analyzed the public’s environmental awareness and its influence on their WTP for haze governance. 

There have been various studies of WTP for haze reduction in China, including at the city level, such as Zibo and Jinan [10,11]; directly-controlled municipalities, including Chongqing [12], Beijing, and Shanghai [13]; the provincial level, such as Shandong and Fujian provinces [13]; regional areas, such as Beijing-Tianjin-Hebei [14] and the Yangtze River Delta [15]; and at the national level [16]. Zhou J et al. found that people in different cities had different WTP values for haze improvement in 2007 [17]. But, there is a research gap caused by the lack of comparative intercity studies assessing WTP levels across different cities within a single Chinese province. The lack of intra-province differences in WTP is surprising because environmental policy is mainly developed and implemented at the province level. Yet, policy makers have proceeded to design and implement policy lacking knowledge of any intercity WTP differences or the factors that might account for any such WTP differences. To ensure that haze governance projects are consciously endorsed and supported, policymakers need to address any intercity differences in public attitudes towards haze reduction, especially whether or not the public is willing to share the cost of smog governance. Only through a better understanding of any intercity differences in public preferences towards haze improvement, and securing public support for haze reduction policies, will sustainable provincial and national environmental policies, with their attendant health and social well-being benefits, be achieved.

## 2. Study Design

### 2.1. Study Site, Sampling Technique, and Data Collection 

Our study site was Shandong province, a major east coast heavy industry province experiencing rapid industrialization and urbanization, with a GDP that was ranked third after Guangdong and Jiangsu and a per capita GDP that was ranked tenth in China in 2015 [18]. With a mix of chemical, auto, food processing, engineering, and machinery industries, coal consumption accounts for around 80% and crude oil consumption around 15% of Shandong’s total energy consumption [18]. A combination of stratified sampling and non-probability sampling methods were used to collect the data. First, Shandong province’s 17 cities were stratified into four clusters in terms of indicators comprising per capita GDP, a ratio of the income from the secondary sector to GDP, net income of rural residents, disposal income of urban residents, the number of private cars owned, and the number of heavy polluted days over a year. Second, a city was randomly selected in each stratification, yielding Jinan, Yantai, Zibo, and Linyi as our sampling cities. Face-to-face interviews with 1033 residents aged more than 18 years old were conducted at random in November 2014 at places attracting large crowds, such as supermarkets, squares, and gardens. There were 1006 usable questionnaires, with a valid response rate of 97.39%. Face-to-face interviews have the advantage of ensuring good response rates to all questions and the exchange of information to reduce misunderstanding or misinterpreting the survey questions. The sample sizes in each city are presented in Table 1. 

**Figure 1:**
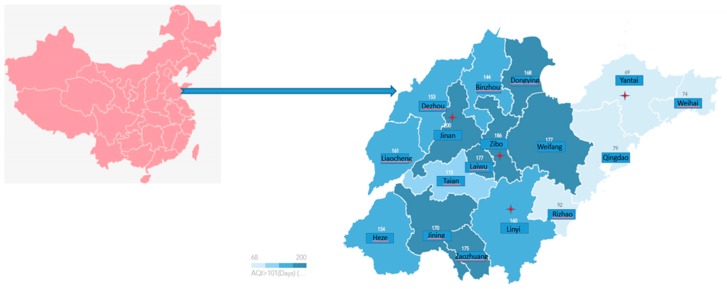
Air Quality Index > 101 days in Shandong (2014).

Air quality varied across the four cities. As shown in detailed air quality measures in Table 1 and Figure 1, the 2014 November air quality in Yantai was the best among the four cites, with Jinan in the middle and Zibo and Linyi being the worst. These intra-provincial differences reflected the industrial mix of the four cities and seasonality factors. Shandong’s sizable agricultural sector meant that the traditional custom of burning-off the wheat or corn straw produced significant smoke in the harvest seasons and during winter, there was a significant rise in air pollution due to central coal heating. Considering these seasonality factors, we undertook the investigation in November. 

### 2.2. Questionnaire and the CVM Survey

A survey questionnaire addressed residents’ attitude and behavior concerning haze governance in the four cities. The questionnaire consisted of four parts: (1) the residents’ socioeconomic status, including gender, age, education, marital status, region of residence, occupation, and monthly income level; (2) the residents’ knowledge and behavior regarding haze; (3) the residents’ satisfaction and support for haze governance policies; and (4) the residents’ WTP for improving the haze situation.

**Figure 2:**
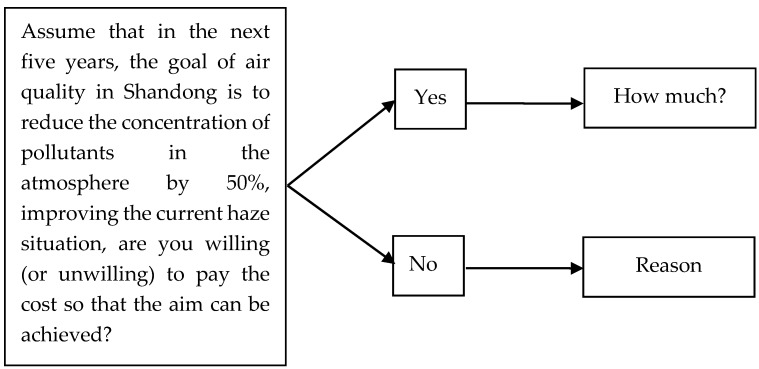
Main structure of the CVM survey.

We employed the contingent valuation method (CVM) [19,20,21,22], which presents consumers with hypothetical opportunities to buy public goods, where the WTP for a given commodity is elicited directly through a survey, thus circumventing the absence of a real market for public goods [23]. We selected a realistic payment option in the CVM survey [24]. Respondents were presented with a hypothetical situation that provided full information on the haze problem, allowing them to reveal their values as accurately as possible. Figure 2 presents the diagrammatic of the open-ended hypothetical valuation questions. Respondents that were willing to pay, were then asked: “How much are you willing to pay?”. Those who chose “unwillingness to pay” were asked for their reasons. To avoid any spurious emotions affecting responses, they were informed that the study was being carried out for academic purposes only.

### 2.3. Measurement of Haze Attitudes

#### 2.3.1. The Environmental Awareness and Confidence Indicators

To assess respondents’ haze understanding and haze behavior, an environmental awareness indicator was constructed. The indicator consisted of two parts: respondents’ subjective perception of haze weather and respondents’ objective concern and knowledge of haze weather. In the construction of the index, we used the Delphi method to consult 20 experts in the public health and environmental health area across five Chinese universities. As shown in Table 2, each index comprised four questions, measured by five-point and two-point severity scales. Aggregating the four subjective haze questions, the index ranged between 4 (lowest concern) and 20 (highest concern) points, where residents’ subjective perception of haze weather was categorized as 4–5 very good, 6–10 good, 11–15 bad, and 16–20 very bad. The objective indicators of residents’ knowledge of haze weather ranged from 2 to 12, with 2–3 labeled not concerned/understand, 4–6 little concern/understanding, 7–9 moderate concern/understanding, and 10–12 high concern/understanding.

#### 2.3.2. Confidence and Satisfaction in Government Indicator

Table 3 displays the five-point satisfaction scale for five questions measuring residents’ confidence and satisfaction in central and local government haze governance. 

The government confidence and satisfaction indicators aggregated from 5 (lowest confidence in government) to 25 (highest confidence in government) points, which were then classified into four confidence/satisfaction with government groups: 5–10 no confidence/satisfaction; 11–15 neutral; 16–20 confidence/satisfaction; and 21–25 high confidence/satisfaction.

### 2.4. Statistical Tools and Analytical Models

Survey-based data often have to cope with missing data. In our CVM survey, many respondents were unwilling to pay for improving the haze situation. Therefore, we lost these bidders when referring to the amount of cash WTP respondents selected. Further, high bidders may have observed and unobserved attributes. Specifically, higher income, younger, better educated respondents may be more likely to pay and to pay more for haze improvement than other respondents. Also, those who are willing to pay more for haze reduction may have stronger attitudes towards purchasing for air quality. Hence, the analysis of the determinants of respondents’ WTP amount may potentially suffer from attitude-based selection bias. Specifically, parameter estimates of the determinants of the WTP (attitude) may be biased. One way we selected to control for this attitude-based selection bias was to jointly estimate the determinants of WTP and WTP amount.

Following the Heckman sample selection model [25], the latent relationship between respondents’ attributes and choice of paying for improving the haze situation is modeled as:

Z_i_* = *W_i_*’*γ* + *μ_i_* (1)

where Z_i_* is the latent measure of an individual’s WTP, *W_i_*’ is a vector of characteristics for individual *i*, *γ* is the corresponding vector of coefficients to be estimated, and *μ_i_* is the random disturbance for individual *i* [26]. The observed outcome is:

(2) $$\{\begin{array}{l} Z_{i}=1,\mathrm{if}\;Z_{i}^{*}>0\\ Z_{i}=0,\;\mathrm{if}\;Z_{i}^{*}\leq 0 \end{array}$$

However, information on the amount of WTP is only available if the purchase decision is reported by the respondents. Respondents’ latent purchase amount is modeled as:

Y_i_* = *X_i_*’*β* + *ε_i_* (3)

where *Y_i_* is the dependent variable (the observed realization of another latent variable Y_i_*), which is observable if and only if Z_i_* exceeds a certain threshold. In our case, *Y_i_* is the non-zero price elasticity of demand for haze improvement, *X_i_*’ is a vector of covariates for individual *i*, *β* is a vector of coefficients for the outcome equation, and *ε_i_* is a random disturbance term for individual *i*. The observed response to the question on “How much is you willing to pay?” is:

(4) $$\{\begin{array}{c} Y_{i}=Y_{i}^{*},\mathrm{if}\;Z_{i}\neq 0\;\\ Y_{i}=\mathrm{missing},\;\mathrm{if}\;Z_{i}=0 \end{array}$$

Estimation of WTP choice with the sub-sample who provide a positive response on haze improvement is equivalent to:

*E*(*Y*|Z_i_* > 0) = *E* (*X_i_*’*β* + *ε_i_*|*W_i_*’*γ* + *μ_i_* > 0) = *E* (*X_i_*’*β* + *ε_i_*/*μ_i_* > *W_i_*’*γ*) = *X_i_*’*β* + *E* (*ε_i_*/*μ_i_* > −*W_i_*’*γ*) = *X_i_*’*β* + *ρσελ*(−*W_i_*’*γ*) (5)

Assume *E* (*ε_i_*) = *E* (*μ_i_*) = 0, and *ρ* = corr (*ε_i_*, *μ_i_*) 

The model assumed that the error terms have a bivariate normal distribution with zero means and correlation rho (*ρ*), where the significance of the *ρ* (*ρ* = 0) was used to infer that Z* and Y* are correlated, and there was a sample selection problem. In our case, a significant rho statistic indicates whether there are unobservable factors affecting an individual’s WTP that were correlated with the price sensitivity of haze attitude.

Statistical packages Stata12.0 (StataCorp LP., College Station, TX, USA) and SPSS 17.0 (SPSS Inc., Chicago, IL, USA) were employed to estimate the models.

## 3. Results

Table 4 reports the descriptive statistics for the main socio-economic characteristics and haze awareness and confidence of the sample respondents in the four cities. The respondents were evenly distributed between male (51.6%) and female (48.4%), with a mean age of 37 years, and slightly skewed towards the younger age groups, especially for Linyi. For the income level, over 50% of respondents earn less than US$465.84 (CNY3000) per month, which is lower than the US$670.61 (CNY4318.75) average monthly wage level in Shandong province (see Table 4). In terms of marital status, 70% of the respondents were single, reflecting the young age distribution of the sample. With 99% respondents educated, Table 4 shows that the respondents in Yantai were better educated than the other cities, with over 80% respondents having at least a high school education. About 50% of respondents were office workers or public servants, 17% were self-employed, and 11% were students, reflecting the bias toward urban white-collar workers, which also explains the lower than average Shandong monthly wage, especially in Linyi. In short, the respondents were more likely to be young and educated, but with a lower monthly income than the average Shandong resident.

As for haze awareness and confidence, there were significant variations in haze attitudes across the four cities (at *p* = 0.05 level). In Table 4, Jinan had the largest percentage of respondents with a “bad” (91%) subjective haze assessment and “no confidence and satisfaction” (16%) in the government, compared to about 72% with a “bad” subjective haze assessment in the other cities. Zibo had the largest percentage (26%) of respondents who had “high concern” with haze and Yantai had the lowest percentage (14%) with a “high concern” with haze.

In Table 5, using the Heckman model, we analyzed the difference in WTP amount across the four cities and in Table 6, we presented the socio-economic characteristics and haze attitudes toward people with positive WTP. As shown in Table 5, Rho (*ρ* = 0) was significant, which meant that the WTP amount was associated with several characteristics that affect their WTP. Specifically, there were significant differences in WTP across the four cities: Yantai, Zibo, and Linyi were more willing to pay for haze improvement than Jinan residents (at the *p* = 0.001 level). Further, Table 5 shows the common patterns found in previous studies related to age, trust of government, haze attitudes, income, education, and haze knowledge. Respondents’ WTP amount was negative for those over 40 years old, but positive for 18–30-year-old respondents, and also negative for those with incomes below US$155.28 (CNY1000). 

Table 6 displays the details of the WTP across the four cities: 55.51% respondents in Linyi were willing to pay for haze governance, followed by Yantai (48.56%), Zibo (51.12%), and Jinan (33.33%). Respondent age was a significant factor (test statistic χ^2^ = 26.42, *p*-value < 0.05) in the willingness to pay for haze improvement, with over 60% of the respondents under 40 years old willing to pay for haze improvement. Importantly, different age groups across the four cities had a different WTP. Further, respondents in different income levels (χ^2^ = 31.61, *p*-value < 0.001) had a significantly different WTP across the four cities. As shown in Table 6, there were also significantly different subjective indicators across the four cities, with 93.83% respondents in Jinan feeling “bad” about the haze situation compared to 73.73% in Yantai, 74.26 in Zibo, and 78.01% in Linyi.

The major reasons for respondents’ unwillingness to pay for haze improvement are presented in Table 7. About two thirds of the non-payers argued that air quality improvement was mainly the responsibility of governments (39%) and polluters (25%), instead of ordinary citizens, but 27% said that their income was too low to contribute to a pollution tax and 6% claimed that they did not believe the funds would be used effectively for environmental conservation. There were significant differences across cities. Non-payers in Zibo (43.85%) Jinan (42.50%), and Yantai (41.94%) thought the government should pay for haze reduction, but a significantly lower percentage of Linyi respondents thought this (26.55%). Linyi had a significantly larger proportion of respondents (35.40%) who thought polluters should pay for haze reduction. In terms of income being too low to afford haze reduction, Zibo had the largest proportion of non-payers (33.08%), followed by Jinan (30.63%) and Linyi (29.20%), with Yantai (15.32%) having the lowest proportion. Yantai (11.29%) and Linyi (7.96%) were significantly more concerned than the other cities on how haze reduction funds might be effectively used. 

As shown in Table 8, there were significant variations in non-payer’s socio-economic characteristics and haze attitudes across the four cities. Jinan had the largest percentage of non-payers, with respondents being older and less educated, and having a “bad” perception of the haze situation and less confidence in the government compared to respondents in the other cities. Specifically, about 50% non-payers in Jinan were over the age of 40, and 38.27% did not have a high school or more advanced education. Jinan had the largest number of non-payers feeling bad about the haze situation.

## 4. Discussion

Given its industrial structure and rural-urban composition, the four cities studied in Shandong are typical of industrializing China [27,28]. Therefore, our analysis and results are comparable to other parts of transitioning China. In each city, an overwhelming majority of respondents were concerned about haze pollution, but only a minority of respondents were willing to pay for haze governance, and, for those WTP, the WTP amount varied across the four cities due to age and income. These differences were significant. 

The socio-economic structure of each city had an impact on the WTP. For example, age interacted with respondents’ income and education level, with older, poorer, less educated, and less environmentally aware respondents less willing to pay and less willing to pay large amounts for haze reduction. Linyi had the highest WTP respondents under the age of 40, while Jinan had the highest WTP respondents over the age of 40 among the four cities, which may indicate that positive WTP in different cities reflected different age distributions, so policy makers need to consider different age distributions when setting environmental policy.

Second, income had a positive impact on WTP for haze governance, but WTP varied across the four cities by income level. Only 15.32% of non-payers in Yantai reported that their income was too low to afford haze reduction levies, representing about half the percentage of the other three cities. 

Third, respondents’ subjective feelings toward their local haze differed across the four cities. Jinan had the lowest WTP respondents among the four cities (33.33%), but the largest proportion of WTP respondents who felt “bad” (93.83%) about the local haze situation. The other three cities had about 50% WTP respondents, with 75% who felt “bad” about the haze weather. These subjective factors suggest complex motivations for the WTP and WTP amount: those in self-identifying bad haze environments, such as Jinan, but with objective better haze environments (see Table 1), might pay less for haze reductions than those in Zibo and Linyi, with better self-assessed, but worse objective, haze environments. So different cities with different subjective haze ratings might support haze reduction policies for very different reasons, which policy makers should take into consideration when setting and promoting haze reduction policies. 

This also applies for differences in respondents’ satisfaction with the government’s haze reduction credentials. Linyi had the largest proportion of WTP respondents with “confidence” in the government, while Jinan had the largest proportion with a “neutral” belief in the government, which suggests that people in different cities placed a different reliance on the local and provincial government for haze governance, and thus displayed a different WTP. One of the main reasons respondents gave for their unwillingness to pay was their belief that haze governance was mainly the responsibility of governments (39.3%) and polluters (25.6%) [1]. Linyi had the lowest proportion (26.55%) of non-payers who thought haze reduction should be paid by the government, and the highest proportion (35.40%) who believed that haze reduction should be paid by polluters. Across the four cities, two contradictory factors were at work: some respondents’ distrust of the government to manage haze governance versus the high expectation from other respondents that the government will address the haze problem. Policy makers need to consider the different attitudes to the government when making environmental policy. By placing the responsibility on the government, unwilling to pay respondents believed that they had paid sufficient taxes and that polluters needed to pay to reduce haze. Our data suggest that the government failed to persuade a significant proportion of the residents in certain cities that payment for haze abatement was a public, rather than a government or polluter, problem. 

WTP in our survey was significantly lower than the positive WTP for haze governance in previous studies of Chongqing (62%) and 59.7% in Jinan [11,12]. Some of the difference might be due to our use of open-ended questions to ask for respondents’ WTP amounts instead of the bidding game used in the Chongqing study [12]. Respondents may be more or less sensitive about the price of public goods depending on the question format. Further, Probit models used in previous studies do not easily control for sample selection bias compared to our use of the Heckman selection model. Different WTP amounts in previous studies also reflect different sample characteristics, with our respondents being urban, young, and well educated, with more students and lower paid white-collar workers [11,12]. 

Income was an important factor in all previous studies of WTP for haze reduction [10,12,29,30]. In contrast to Huichen Xian and Hu Meng’s study, where awareness of air quality was more important than income in determining WTP, income was the most important determinant of WTP in our model [31]. But, our results show WTP diversity by income across the four cities, so the income factor was mediated by haze awareness and other factors. Our analysis revealed that environmental awareness, including subjective perception and objective behaviors, and satisfaction with the government, had a significant impact on respondents (un)willing to pay. Subjective indicators exerted a negative impact on WTP. Objective indicators and satisfaction with the government exerted a positive impact on respondents’ willingness to pay. Our results are consistent with other studies which showed that the public’s environmental awareness increases their willing to pay for protecting the environment [32,33].

Like all empirical studies, our WTP findings need careful interpretation. First, the results are based on an urban sample. The results for a sample including rural and industrial workers might be different, but are likely to be biased towards lower WTP for haze reduction due to lower education, incomes, and knowledge of environmental issues. Second, clean air is a pure public good, which means that there is a free-rider problem. Our respondents’ education level and knowledge of environmental problems may mean that they are aware of the free-rider problem, which decreased their WTP. Also, we should be aware that the WTP based on CVM might not be the same as actual mean WTP [34]. Future studies might employ other CVM techniques, such as discrete-choice models, and alternative approaches, such as payment card methods [35]. 

## 5. Conclusions

Haze governance is a major aim and challenge for the Chinese government’s environmental policies, which address the health and well-being of China’s population. Our study investigated the haze attitudes and the WTP for haze governance in four industrial cities in Shandong province. WTP and WTP amount varied by city. The factors driving these differences in WTP and WTP amounts, such as age, income, education, and subjective and objective factors, also differed across the four cities. Finally, the main unwilling to pay reason, the belief that haze governance should be the responsibility of governments and the polluters, also varied across the four cities. 

In conclusion, the city-specific differences in WTP may caution against “one size fits all” policies. Broad-based education campaigns, for example, are likely to reach only income, education, and occupational groups already convinced about the pubic good nature of the haze problem. Tailoring policies to specific cities and the characteristics of residents in those cities poses significant challenges to policy making. Targeting subgroups of the city population will require the government to collect more data on the socio-economic factors shaping individual residents characteristics and haze improvement attitudes. This is a daunting task for policy makers and policy implementers. But only by reaching income, age, and education subgroups within a city can environmental policy convince the population to contribute to paying for clean air. China’s national level policy pronouncements to bring “blue skies” will depend on how subgroups of residents in individual cities perceive environmental policies. Collecting city-specific subgroup data on environmental attitudes and understanding is the first step to designing targeted interventional strategies to address the haze problem in China. 

## Figures and Tables

**Table 1:** Sample sizes and air quality of the four cities in Shandong province (among 17 cities in November, 2014).

City	Population (million)	Area (km^2^)	GDP per Capita (US$)	Average Concentration of Nitrogen Dioxide (μg/m^3^)		Average Concentration of PM_10_ (μg/m^3^)		Average Concentration of PM_2.5_ (μg/m^3^)		Days of Visibility over 10 m	
				Value	No.	Value	No.	Value	No.	Value	No.
Yantai	6.64	13,746	12,477.79	48	3	76	1	60	2	22	3
Jinan	5.92	8177	11,645.03	64	11	172	11	100	7	9	10	
Zibo	4.18	5965	12,870.96	74	15	187	14	120	14	8	15
Linyi	9.94	17,184	5109	74	16	189	15	115	13	8	16

Source: Shandong Environmental Protection Department.

**Table 2:** Environmental awareness indicators.

Subjective Indicators	Options of Response	Objective Indicators	Options of Response
How would you rate the haze weather situation in your locality?	Extremely serious = 5 Very serious = 4 Moderately serious = 3 A little serious = 2 Not serious = 1	Do you know what haze is?	Yes = 1 No = 0
Were there times when you experience haze pollution in your locality?	Always = 5 Often = 4 Sometimes = 3 Rarely = 2 Never = 1	Do you know the causes of haze weather?	Totally = 5 A lot = 4 Moderately = 3 A little = 2 Not at all = 1
How would you evaluate air quality in the fall and winter last year?	Very poor = 5 Poor = 4 Fair = 3 Good = 2 Very good = 1	Have you ever sought knowledge on health protection during haze weather?	Yes = 1 No = 0
How would you rate the influence of haze weather on your daily life?	Major impact = 5 large impact = 4 medium impact = 3 small impact = 2 no impact = 1	Do you pay attention to your local air quality index?	Yes, completely = 5 Yes, mostly = 4 Yes, a little = 3 No, not really = 2 No, not at all = 1

**Table 3:** Confidence and satisfaction in government indicators.

	The Variables	Options of Response
1	Satisfied with the haze monitoring and prediction work of the central government	Highly satisfied = 5 Satisfied = 4 Neutral = 3 A little dissatisfied = 2 Not satisfied = 1
2	Satisfied with the haze control work of the central government	
3	Satisfied with the haze monitoring and prediction work of the local government	
4	Satisfied with the haze control work of the local government	
5	The confidence of effectiveness of government policies about haze governance	Totally confident = 5 Confident = 4 Neutral = 3 A little unconfident = 2 Highly unconfident = 1

**Table 4:** Socio-economic characteristics and haze attitudes of the respondents (*N* = 1006).

Variables	Group	Proportion of Total (%) (*N* = 1006)	Jinan (%) (*N* = 243)	Yantai (%) (*N* = 243)	Zibo (%) (*N* = 266)	Linyi (%) (*N* = 254)	χ^2^ (*p*-Value)
Gender	male	51.6	51.9	48.6	50.2	55.9	3.064(0.382)
	female	48.4	48.1	51.4	50.2	44.1	
Age	18–29	38.5	32.5	34.6	35.3	51.2	73.650(0.000)
	30–39	22.2	20.2	28.0	20.3	20.9	
	40–49	19.1	22.2	14.0	18.8	21.2	
	50–59	14.1	18.9	16.9	15.4	5.5	
	≥60	6.1	6.2	6.5	10.2	1.2	
Income (monthly, CNY)	≤1000	18.6	15.6	17.7	14.3	26.8	29.709(0.003)
	1000–2999	41.1	39.1	39.1	49.2	36.2	
	3000–4999	32.2	37.9	33.3	28.2	29.9	
	5000–7999	6.1	4.9	6.6	6.0	6.7	
	≥8000	2.1	2.5	3.3	2. 3	0.4	
Marriage status	married	30.4	23.5	31.7	25.6	40.9	22.009(0.000)
	single	69.6	76.5	68.3	74.4	59.1	
Education level	below primary school	0.8	0.4	0.8	0.0	2.0	37.014(0.000)
	primary/middle school	25.7	31.7	16.1	25.6	29.5	
	high school	33.9	35.4	34.2	37.2	28.7	
	college	38.7	30.9	47.7	37.2	39.0	
	master or above	0.9	1.6	1.2	0.0	0.8	
Occupation	civil servant, public institution	10.5	9.9	12.4	6.8	13.4	128.647(0.000)
	office staff	38.7	40.3	39.1	44.0	31.1	
	self-employed	17.4	19.3	15.2	13.9	21.3	
	worker and peasant	5.6	6.6	3.7	6.4	5.5	
	student	10.6	8.2	9.5	3.4	21.6	
	freelance work	2.8	9.1	9.1	14.7	1.2	
	retired people	8.5	4.1	4.9	0.7	1.5	
	unemployed	3.6	1.2	2.5	8.3	2.0	
	others	2.3	1.2	3.7	1.9	2.3	
Subjective indicator	very good	2.2	0.0	3.7	1.9	3.2	43.326(0.000)
	good	20.3	8.2	21.8	26.3	24.0	
	bad	77.0	90.9	73.7	71.4	72.8	
	very bad	0.5	0.8	0.8	0.4	0.0	
Objective indicator	no concern	2.7	1.7	2.1	4.5	2.4	20.963(0.013)
	little concern	30.8	29.6	34.9	25.6	33.5	
	moderate concern	45.8	43.2	49.0	43.6	47.6	
	high concern	20.7	25.5	14.0	26.3	16.5	
Confidence and satisfaction in government indicator	no confidence	12.8	16.1	12.4	12.4	10.6	30.518(0.000)
	neutral	34.6	44.4	31.7	33.5	29.1	
	confidence	41.2	31.7	48.2	41.0	43.7	
	high confidence	11.3	7.8	7.8	12.8	16.5	
Total		100.0	24.2	24.2	26.4	25.2	

**Table 5:** Heckman sample selection model analysis (*N* = 1006). (Wald Chi^2^ = 70.37, *P* = 0.000).

Independent Variables		(1) Outcome Part Log (WTP amount)		(2) Selection Part WTP	
		Parameter Estimate	*p*-Value	Parameter Estimate	*p*-Value
Social-economic information					
Age (1 = 18–30)	2 = 30–40	−0.785	0.276	−0.255 *	0.091
	3 = 40–50	−1.046 **	0.049	−0.193	0.155
	4 = 50–60	−1.393 **	0.032	−0.260	0.180
	5 = over 60	−0.842	0.358	−0.089	0.267
City (1 = Jinan)	2 = Yantai	0.909	0.124	0.421 ***	0.005
	3 = Zibo	0.833	0.799	0.570 ***	0.003
	4 = Linyi	0.743	0.812	0.500 ***	0.000
Gender (1 = female)	2 = male	−0.266	0.313	−0.104	0.252
Occupation (1=civil servant, public institution)	2 = office staff	−0.274	0.216	−0.017	0.913
	3 = self-employed	0.031	0.124	0.295 *	0.000 ***
	4 = worker and peasant	−0.326	0.217	0.072	0.102
	5 = student	1.859	0.124	0.810 ***	0.000
	6 = freelance work	−0.637	0.569	−0.215	0.351
	7 = retired people	−0.087	0.167	0.353	0.283
	8 = unemployed	0.146	0.783	0.024	0.997
	9 = others	0.152	0.290	0.545	0.789
Monthly income (1 = below 1000)	2 = 1000–2999 3 = 3000–4999 4 = 5000–7999 5 = over 8000	1.426	0.218	0.473 **	0.020
		2.191	0.122	0.689 ***	0.002
		1.857 **	0.032	0.315	0.191
		1.513	0.290	0.626*	0.089
Marriage (1 = single)	2 = married	0.177	0.340	0.006	0.496
Education (1 = below primary school)	2 = primary/middle school	0.858	0.506	1.158 **	0.037
	3 = high school	0.560	0.415	1.039 *	0.062
	4 = college	0.283	0.323	0.926	0.101
	5 = master or above	0.168	0.186	1.154	0.154
Environmental awareness and confidence indicators					
Objective indicators (1 = not concern)	2 = little concern	−0.093	0.130	0.644 **	0.036
	3 = moderate concern	0.442	0.400	0.752 **	0.016
	4 = high concern	1.004	0.737	0.951 ***	0.003
Confidence and satisfaction in government indicators (1 = no confidence)	2 = neutral confidence	0.389	0.720	0.208	0.140
	3 = confidence high	0.523	0.721	0.423 ***	0.002
	4 = confidence	1.004	0.773	0.680 ***	0.000
Subjective indicators (1 = Very good)	2 = good	−0.064	0.363	0.349	0.247
	3 = bad	−0.065	0.214	0.470	0.121
	4 = very bad	1.804	0.891	1.494 **	0.046
Constant		−0.285	0.128	−1.345 *	0.057
Rho (*ρ* = 0)		Chi^2^ = 5.21		*P* = 0.0225	

Note: * indicate significance at the *p* = 0.10 level, ** indicate significance at the *p* = 0.05 level, *** indicate significance at the *p* = 0.001 level.

**Table 6:** Socio-economic characteristics and haze attitudes toward people with positive WTP.

Variables	Jinan (*n* = 81)	Yantai (*n* = 118)	Zibo (*n* = 136)	Linyi (*n* = 141)	χ^2^	*ρ*
Gender						
Male	44 (54.32%)	57 (48.31%)	67 (49.26%)	72 (51.06%)	0.80	0.849
Female	37 (45.68%)	61 (51.69%)	69 (50.74%)	69 (48.94%)		
Age						
<30	35 (43.21%)	48 (40.68%)	60 (44.12%)	81 (57.45%)	26.42	0.009
30–40	18 (22.22%)	32 (27.12%)	29 (21.32%)	29 (20.57%)		
40–50	16 (19.75%)	15 (12.71%)	24 (17.65%)	25 (17.73%)		
50–60	10 (12.35%)	13 (11.02%)	13 (9.56%)	6 (4.26%)		
>60	2 (2.47%)	10 (8.47%)	10 (7.35%)	0 (0.00%)		
Income						
<1000	13 (16.05%)	23 (19.49%)	12 (8.82%)	43 (30.50%)	31.61	0.002
1000–2999	29 (35.80%)	40 (33.90%)	65 (47.79%)	50 (35.46%)		
3000–4999	35 (43.21%)	45 (38.14%)	45 (33.09%)	42 (29.79%)		
5000–7999	2 (2.47%)	7 (5.93%)	9 (6.62%)	6 (4.26%)		
>8000	2 (2.47%)	3 (2.54%)	5 (3.68%)	0 (0.00%)		
Education						
below primary school	0 (0.00%)	2 (1.69%)	0 (0.00%)	4 (2.84%)	17.81	0.122
primary/middle school	16 (19.75%)	16 (13.56%)	28 (20.59%)	37 (26.24%)		
high school	29 (35.80%)	37 (31.36%)	53 (38.97%)	38 (26.95%)		
college	35 (43.21%)	62 (52.54%)	55 (40.44%)	61 (43.26%)		
master or above	1 (1.23%)	1 (0.85%)	0 (0.00%)	1 (0.71%)		
Marriage status						
married	27 (33.33%)	42 (35.59%)	39 (28.68%)	62 (43.97%)	7.32	0.062
single	54 (66.67%)	76 (64.41%)	97 (71.32%)	79 (56.03%)		
Occupation						
civil servant, public institution	6 (7.41%)	16 (13.56%)	10 (7.35%)	19 (13.48%)	65.45	0.000
office staff	33 (40.74%)	43 (36.44%)	64 (47.06%)	37 (26.24%)		
self-employed	18 (22.22%)	19 (16.10%)	23 (16.91%)	29 (20.57%)		
worker and peasant	3 (3.70%)	4 (3.39%)	9 (6.62%)	7 (4.96%)		
student	12 (14.81%)	15 (12.71%)	6 (4.41%)	38 (26.95%)		
freelance work	4 (4.94%)	9 (7.63%)	16 (11.76%)	1 (0.71%)		
retired people	5 (6.17%)	4 (3.39%)	1 (0.74%)	3 (2.13%)		
unemployed	0 (0.00%)	3 (2.54%)	4 (2.94%)	3 (2.13%)		
others	0 (0.00%)	5 (4.24%)	3 (2.21%)	4 (2.84%)		
Subjective indicator						
very good	0 (0.00%)	2 (1.69%)	2 (1.47%)	3 (2.13%)	21.29	0.011
good	3 (3.70%)	28 (23.73%)	32 (23.53%)	28 (19.86%)		
bad	76 (93.83%)	87 (73.73%)	101 (74.26%)	110 (78.01%)		
very bad	2 (2.47%)	1 (0.85%)	1 (0.74%)	0 (0.00%)		
Objective indicator						
not concern	0 (0.00%)	2 (1.69%)	3 (2.21%)	2 (1.42%)	15.64	0.075
little concern	16 (19.75%)	36 (30.51%)	32 (23.53%)	44 (31.21%)		
moderate concern	35 (43.21%)	57 (48.31%)	63 (46.32%)	70 (49.65%)		
high concern	30 (37.04%)	23 (19.49%)	38 (27.94%)	25 (17.73%)		
Confidence and satisfaction in government indicator						
no confidence	7 (8.64%)	12 (10.17%)	18 (13.24%)	7 (4.96%)	12.42	0.191
neutral	30 (37.04%)	39 (33.05%)	40 (29.41%)	37 (26.24%)		
confidence	33 (40.74%)	53 (44.92%)	62 (45.59%)	69 (48.94%)		
high confidence	11 (13.58%)	14 (11.86%)	16 (11.76%)	28 (19.86%)		
N	243	243	266	254		
n/N (%)	33.33%	48.56%	51.12%	55.51%		
WTP amount(mean)	CNY72.12	CNY95.96	CNY83.99	CNY104.64		

**Table 7:** Respondents’ reasons for not paying.

Reasons for not Paying	Jinan (%) (*n* = 160)	Yantai (%) (*n* = 124)	Zibo (%) (*n* = 130)	Linyi (%) (*n* = 113)	χ^2^	*p*
Should be paid by government	42.50	41.94	43.85	26.55	46.50	0.000
Income is too low to afford it	30.63	15.32	33.08	29.20		
Who pollute the environment should pay	23.75	29.84	15.38	35.40		
Worry about whether funds would be used for environment conservation effectively	3.13	11.29	3.85	7.96		
Air quality is not bad	0.00	1.61	1.54	0.88		
Other reasons	0.00	0.00	2.31	0.00		
Total	100.00	100.00	100.00	100.00		

**Table 8:** Socio-economic characteristics and haze attitudes toward non-payers.

Variables	Jinan (*n* = 162)	Yantai (*n* = 125)	Zibo (*n* = 129)	Linyi (*n* = 113)	χ^2^	*P*
Gender						
Male	80 (49.38%)	64 (51.20%)	64 (49.23%)	43 (38.05%)	5.11	0.164
Female	82 (50.62%)	61 (48.80%)	66 (50.77%)	70 (61.95%)		
Age						
<30	53 (32.72%)	44 (35.20%)	40 (30.77%)	59 (52.21%)	53.43	0.000
30–40	29 (17.90%)	38 (30.40%)	23 (17.69%)	29 (25.66%)		
40–50	43 (26.54%)	18 (14.40%)	31 (23.85%)	23 (20.35%)		
50–60	29 (17.90%)	21 (16.80%)	21 (16.15%)	1 (0.88%)		
>60	8 (4.94%)	4 (3.20%)	15 (11.54%)	1 (0.88%)		
Income						
<1000	25 (15.43%)	20 (16.00%)	26 (20.00%)	25 (22.12%)	14.79	0.253
1000–2999	66 (40.74%)	55 (44.00%)	66 (50.77%)	42 (37.17%)		
3000–4999	57 (35.19%)	36 (28.80%)	30 (23.08%)	34 (30.09%)		
5000–7999	10 (6.17%)	9 (7.20%)	7 (5.38%)	11 (9.73%)		
>8000	4 (2.47%)	5 (4.00%)	1 (0.77%)	1 (0.88%)		
Education						
below primary school	1 (0.62%)	0 (0.00%)	0 (0.00%)	1 (0.88%)	21.52	0.043
primary/middle school	61 (37.65%)	23 (18.40%)	40 (30.77%)	38 (33.63%)		
high school	57 (35.19%)	46 (36.80%)	46 (35.38%)	35 (30.97%)		
college	40 (24.69%)	54 (43.20%)	44 (33.85%)	38 (33.63%)		
master or above	3 (1.85%)	2 (1.60%)	0 (0.00%)	1 (0.88%)		
Marriage status						
married	132 (81.48%)	90 (72.00%)	101 (77.69%)	71 (62.83%)	13.30	0.004
single	30 (18.52%)	35 (28.00%)	29 (22.31%)	42 (37.17%)		
Occupation						
civil servant, public institution	18 (11.11%)	14 (11.20%)	8 (6.15%)	15 (13.27%)	79.71	0.000
office staff	65 (40.12%)	52 (41.60%)	53 (40.77%)	42 (37.17%)		
self-employed	29 (17.90%)	18 (14.40%)	14 (10.77%)	25 (22.12%)		
worker and peasant	13 (8.02%)	5 (4.00%)	8 (6.15%)	7 (6.19%)		
student	8 (4.94%)	8 (6.40%)	3 (2.31%)	17 (15.04%)		
freelance work	18 (11.11%)	13 (10.40%)	23 (17.69%)	2 (1.77%)		
retired people	5 (3.09%)	8 (6.40%)	1 (0.77%)	1 (0.88%)		
unemployed	3 (1.85%)	3 (2.40%)	18 (13.85%)	2 (1.77%)		
others	3 (1.85%)	4 (3.20%)	2 (1.54%)	2 (1.77%)		
Subjective indicator						
very good	0 (0.00%)	7 (5.60%)	3 (2.31%)	5 (4.42%)	33.92	0.000
good	17 (10.49%)	26 (20.80%)	37 (28.46%)	33 (29.20%)		
bad	145 (89.51%)	91 (72.80%)	90 (69.23%)	75 (66.37%)		
very bad	0 (0.00%)	1 (0.80%)	0 (0.00%)	0 (0.00%)		
Objective indicator						
not concern	4 (2.47%)	5 (4.00%)	9 (6.92%)	4 (3.54%)	13.27	0.151
little concern	55 (33.95%)	47 (37.60%)	36 (27.69%)	42 (37.17%)		
moderate concern	71 (43.83%)	60 (48.00%)	55 (42.31%)	50 (44.25%)		
high concern	32 (19.75%)	13 (10.40%)	30 (23.08%)	17 (15.04%)		
Confidence and satisfaction in government indicator						
no confidence	32 (19.75%)	18 (14.40%)	15 (11.54%)	20 (17.70%)	26.37	0.002
neutral	78 (48.15%)	40 (32.00%)	47 (36.15%)	37 (32.74%)		
confidence	44 (27.16%)	60 (48.00%)	52 (40.00%)	42 (37.17%)		
high confidence	8 (4.94%)	7 (5.60%)	16 (12.31%)	14 (12.39%)		
N	243	243	266	254		
n/N (%)	66.67%	51.44%	48.88%	44.49%		

## References

[1] Xue B., Mitchell B., Geng Y., Ren W., Müller K., Ma Z., de Oliveira Jose A.P., Tsuyoshi F., Tobias M. (2014). A review on China’s pollutant emissions reduction assessment. Ecol. Indic..

[2] Geng Y., Fu J., Sarkis J., Xue B. (2012). Towards a national circular economy indicator system in China: An evaluation and critical analysis. J. Clean. Prod..

[3] Xue B., Chen X.P., Geng Y., Yang M., Yang F.X., Hu X.F. (2010). Energy-based study on eco-economic system of arid and semi-arid region: A case of Gansu Province, China. J. Arid Land.

[4] Matus K., Nam K.M., Selin N.E., Lamsal L.N., Reilly J.M., Paltsev S. (2012). Health damages from air pollution in China. Glob. Environ. Chang..

[5] Tao M., Chen L., Xiong X., Zhang M., Ma P., Tao J., Wang Z. (2014). Formation process of the widespread extreme haze pollution over northern China in January 2013: Implications for regional air quality and climate. Atmos. Environ..

[6] Lee S., Oh D.W. (2015). Economic growth and the environment in china: Empirical evidence using prefecture level data. China Econ. Rev..

[7] Huang D., Xu J., Zhang S. (2012). Valuing the health risks of particulate air pollution in the pearl river delta, China. Environ. Sci. Policy.

[8] Xinhuanet. http://www.xinhuanet.com/english/special/2017-11/03/c_136725942.htm.

[9] Reuters. www.Reuters.com.

[10] Wang Y., Sun M., Yang X., Yuan X. (2016). Public awareness and willingness to pay for tackling smog pollution inchina: A case study. J. Clean. Prod..

[11] Wang Y., Zhang Y.S. (2009). Air quality assessment by contingent valuation in Jinan, China. J. Environ. Manag..

[12] Wang H., Mullahy J. (2006). Willingness to pay for reducing fatal risk by improving air quality: A contingent valuation study in Chongqing, China. Sci. Total Environ..

[13] Duan H.X., Yan-Li L., Yan L. (2014). Chinese public’s willingness to pay for CO_2_ emissions reductions: A case study from four provinces/cities. Adv. Clim. Chang. Res..

[14] Xiaoyan L.I. (2016). Empirical analysis of the smog factors in Beijing-Tianjin-Hebei region. Ecol. Econ..

[15] Wang J., Wang S., Voorhees A.S., Zhao B., Jang C., Jiang J., Fu J.S., Ding D., Zhu Y., Hao J. (2015). Assessment of short-term PM_2.5_—Related mortality due to different emission sources in the Yangtze River Delta, China. Atmos. Environ..

[16] Sun C., Yuan X., Yao X. (2016). Social acceptance towards the air pollution in China: Evidence from public’s willingness to pay for smog mitigation. Energy Policy.

[17] Zhou J., Wang Y., Ren L. (2010). Analysis of urban residents’ willingness to pay for improving air quality in typical cities in Shandong Province. J. Environ. Health.

[18] National Bureau of Statistics of China (2015). China Statistical Yearbook 2015.

[19] Mitchell R.C. (1989). Using survey to value public goods. Resour. Futur..

[20] Richard C.B., Patricia A.C., Daniel J.M., Bromley D.W. (1995). Contingent valuation. The Handbook of Environmental Economics.

[21] Sattout E.J., Talhouk S.N., Caligari P.D.S. (2007). Economic value of cedar relics in Lebanon: An application of contingent valuation method for conservation. Ecol. Econ..

[22] Birdir S., Özlem Ü., Birdir K., Williams A.T. (2013). Willingness to pay as an economic instrument for coastal tourism management: Cases from Mersin, Turkey. Tour. Manag..

[23] Whittington D. (2002). Improving the performance of contingent valuation studies in developing countries. Environ. Resour. Econ..

[24] Choongki L., Han S.Y. (2002). Estimating the use and preservation values of national parks’ tourism resources using a contingent valuation method. Tour. Manag..

[25] Heckman J.J. (1979). Sample selection bias as a specification error. Econometrica.

[26] Greene W.H. (2008). Econometric Analysis.

[27] Zhang W. (2010). Shandong: A good example of China in transition. Bus. Week.

[28] Xu X., Gao J., Dockery D.W., Chen Y. (1994). Air pollution and daily mortality in residential areas of Beijing, China. Arch. Environ. Health.

[29] Wang G., Song Y., Chen J., Yu J. (2016). Valuation of haze management and prevention using the contingent valuation method with the sure independence screening algorithm. Sustainability.

[30] Sun C., Yuan X., Xu M. (2014). The public perceptions and willingness to pay: From the perspective of the smog crisis in China. J. Clean. Prod..

[31] Xian H., Hu M. (2013). The study of the residents’willingness to pay for improving air quality in Qingdao. Urban Dev. Stud..

[32] Cuddington J.T., Johnson F.R., Knetsch J.L. (1981). Valuing amenity resources in the presence of substitutes. Land Econ..

[33] Muhammad M., AbulQuasem A.-A., RuliaAkhtar F.K., RafiaAfroz M.R. (2015). Valuing climate protection by offsetting carbon emissions: Rethinking environmental governance. J. Clean. Prod..

[34] Carlson J.L. (2000). Hypothetical surveys versus real commitments: Further evidence. Appl. Econ. Lett..

[35] Bosch J.L., Hammitt J.K., Weinstein M.C., Hunink M.G.M. (1998). Estimating general-population utilities using one binary-gamble question per respondent. Int. J. Soc. Med. Decis. Mak..

